# Dr. Daniel Priory

**Published:** 1859-09

**Authors:** 


DR. DANIEL PRIORY, a physician of much eminence, died of paralysis, at Taunton, on the third of July, in the seventieth year of his age. On account of ill health, he had not practiced his profession for the last twenty years. His contributions to medical literature were voluminous. His first work was published in 1813, under the title of an "Essay on the Absorbents, Comprising some Observations upon the Relative Pathologies and Functions of the Absorbent and Secreting Systems." Two years later, his treatise entitled "A View of the Relations of the Nervous System in Health and Disease" appeared, for which he had gained the Jacksonian Prize. His other works were, "General Indications which Relate to the Laws of Organic Life;" "An exposition of the Principles of Pathology and the Treatment of Diseases;" and "Sketches of Intellectual and Moral Relations." Dr. Priory also contributed largely to medical and scientific journals, and left behind him a large mass of manuscript, which, in a letter to his executor, was ordered to be burned.

